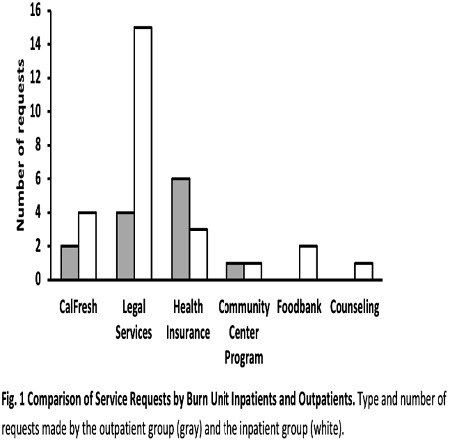# 758 Identifying the Gaps of the Inpatient to Outpatient Transition: The Role of the Patient Navigator

**DOI:** 10.1093/jbcr/irae036.300

**Published:** 2024-04-17

**Authors:** Anika Kim, Cindy Rutter, Daniel W Chacon, Elizabeth Flores, Justin Gillenwater, Karla Anel Gonzalez, Elizabeth Mojarro-Huang, Haig A Yenikomshian

**Affiliations:** Keck School of Medicine at University of Southern California, Los Angeles, CA; 1 Amazing Life, Carlsbad, CA; Alisa Ann Ruch Burn Foundation, San Francisco, CA; Division of Plastic Surgery, University of Southern California Keck School of Medicine, Los Angeles, CA; Keck Medicine of USC, Los Angeles, CA; The Wellness Center at the Historic General Hospital, Los Angeles, CA; Division of Plastic Surgery, University of California Keck School of Medicine, Los Angeles, CA; University of Southern California, Los Angeles, CA; Keck School of Medicine at University of Southern California, Los Angeles, CA; 1 Amazing Life, Carlsbad, CA; Alisa Ann Ruch Burn Foundation, San Francisco, CA; Division of Plastic Surgery, University of Southern California Keck School of Medicine, Los Angeles, CA; Keck Medicine of USC, Los Angeles, CA; The Wellness Center at the Historic General Hospital, Los Angeles, CA; Division of Plastic Surgery, University of California Keck School of Medicine, Los Angeles, CA; University of Southern California, Los Angeles, CA; Keck School of Medicine at University of Southern California, Los Angeles, CA; 1 Amazing Life, Carlsbad, CA; Alisa Ann Ruch Burn Foundation, San Francisco, CA; Division of Plastic Surgery, University of Southern California Keck School of Medicine, Los Angeles, CA; Keck Medicine of USC, Los Angeles, CA; The Wellness Center at the Historic General Hospital, Los Angeles, CA; Division of Plastic Surgery, University of California Keck School of Medicine, Los Angeles, CA; University of Southern California, Los Angeles, CA; Keck School of Medicine at University of Southern California, Los Angeles, CA; 1 Amazing Life, Carlsbad, CA; Alisa Ann Ruch Burn Foundation, San Francisco, CA; Division of Plastic Surgery, University of Southern California Keck School of Medicine, Los Angeles, CA; Keck Medicine of USC, Los Angeles, CA; The Wellness Center at the Historic General Hospital, Los Angeles, CA; Division of Plastic Surgery, University of California Keck School of Medicine, Los Angeles, CA; University of Southern California, Los Angeles, CA; Keck School of Medicine at University of Southern California, Los Angeles, CA; 1 Amazing Life, Carlsbad, CA; Alisa Ann Ruch Burn Foundation, San Francisco, CA; Division of Plastic Surgery, University of Southern California Keck School of Medicine, Los Angeles, CA; Keck Medicine of USC, Los Angeles, CA; The Wellness Center at the Historic General Hospital, Los Angeles, CA; Division of Plastic Surgery, University of California Keck School of Medicine, Los Angeles, CA; University of Southern California, Los Angeles, CA; Keck School of Medicine at University of Southern California, Los Angeles, CA; 1 Amazing Life, Carlsbad, CA; Alisa Ann Ruch Burn Foundation, San Francisco, CA; Division of Plastic Surgery, University of Southern California Keck School of Medicine, Los Angeles, CA; Keck Medicine of USC, Los Angeles, CA; The Wellness Center at the Historic General Hospital, Los Angeles, CA; Division of Plastic Surgery, University of California Keck School of Medicine, Los Angeles, CA; University of Southern California, Los Angeles, CA; Keck School of Medicine at University of Southern California, Los Angeles, CA; 1 Amazing Life, Carlsbad, CA; Alisa Ann Ruch Burn Foundation, San Francisco, CA; Division of Plastic Surgery, University of Southern California Keck School of Medicine, Los Angeles, CA; Keck Medicine of USC, Los Angeles, CA; The Wellness Center at the Historic General Hospital, Los Angeles, CA; Division of Plastic Surgery, University of California Keck School of Medicine, Los Angeles, CA; University of Southern California, Los Angeles, CA; Keck School of Medicine at University of Southern California, Los Angeles, CA; 1 Amazing Life, Carlsbad, CA; Alisa Ann Ruch Burn Foundation, San Francisco, CA; Division of Plastic Surgery, University of Southern California Keck School of Medicine, Los Angeles, CA; Keck Medicine of USC, Los Angeles, CA; The Wellness Center at the Historic General Hospital, Los Angeles, CA; Division of Plastic Surgery, University of California Keck School of Medicine, Los Angeles, CA; University of Southern California, Los Angeles, CA

## Abstract

**Introduction:**

Patient navigators (PNs) are members of the community who help with the transition from inpatient to outpatient care. PNs have demonstrated efficacy in enhancing patient engagement and addressing care gaps in fields such as cancer and vulnerable populations, such as urban, low socioeconomic-status populations. Despite the long-term care needs of burn patients, the role of PNs in the aftercare of burn injury has not been studied. This research investigates the current use of PNs in burn care at a safety-net hospital and sheds light on potential areas of improvement.

**Methods:**

This was a retrospective observational cohort study conducted from November 2022 to August 2023. Data regarding patient contacts, needs identification, and associated referral requisites were collected. The PN role was filled by a representative from a local nonprofit community center. The PN checked in with outpatients after their discharge and inpatients nearing discharge to identify their needs and facilitate appropriate referrals to the relevant community services.

**Results:**

Over eight months, the PN liaised with 658 burn unit patients, comprising 406 outpatients and 252 inpatients. A focused examination of non-COVID services requested reveals that only 3.2% of outpatients requested referrals to community resources. This contrasts with inpatients, where a significantly larger fraction of 10.3% requested similar community service referrals. Figure 1 shows the breakdown of services requested.

**Conclusions:**

While PNs are progressively integrated into various healthcare domains, the newly implemented program in burn care yielded low utilization rates, particularly among outpatients. The disparity between inpatient and outpatient engagement indicates the need to optimize the role of PNs in the transition process from the hospital to outpatient care in burn patients.

**Applicability of Research to Practice:**

The aftercare for burn injuries is complex and extends beyond hospital discharge, requiring effective coordination between various healthcare and community resources. This study highlights the untapped potential of PNs in enhancing outpatient care in the setting of burn injury. Tailoring the PN training to meet the specialized needs of burn patients could enhance their role and improve patient outcomes, particularly for those who face barriers to engagement with the healthcare system.